# A polymorphism in the fatty acid desaturase-2 gene is associated with the arachidonic acid metabolism in pigs

**DOI:** 10.1038/s41598-018-32710-w

**Published:** 2018-09-25

**Authors:** Sofia Gol, Ramona N. Pena, Max F. Rothschild, Marc Tor, Joan Estany

**Affiliations:** 1 Universitat de Lleida - Agrotecnio Center, Department of Animal Science, 25198 Lleida, Catalonia Spain; 2 Iowa State University, Department of Animal Science, Ames, IA 50011 USA

## Abstract

Arachidonic acid (C20:4) is related to a wide range of biological effects including lipid homeostasis. The fatty acid desaturase-2 (*FADS2*) gene encodes for the delta-6-desaturase, which is involved in the biosynthesis of C20:4 from linoleic acid (C18:2). The purpose of this study was to characterise mutations in the promoter of the porcine *FADS2*, evaluating in particular the effect of one haplotype tagging polymorphism (rs321384923A > G) on the biosynthesis pathway of C20:4. A total of 1,192 Duroc barrows with records on fatty acid composition in muscle and subcutaneous fat were genotyped. Pigs carrying the A allele showed, irrespective of fat content, both enhanced *FADS2* expression and higher C20:4 in muscle and exhibited increased ratios of C20:4 to C18:2 and of C20:4 to eicosadienoic acid (C20:2) in both muscle and adipose tissue. Despite the inverse relationship observed between C20:4 and fat content, the rs321384923 polymorphism had no impact on lean weight. It is concluded that the haplotype encompassing the rs321384923 polymorphism at the porcine *FADS2* affects the n-6 fatty acid profile by specifically modifying the desaturation efficiency of C18:2 to C20:4 rather than by concomitant variations in C18:2 following changes in fat content.

## Introduction

Arachidonic acid (C20:4, *all*-*cis*-5,8,11,14–20:4) is the precursor of several bioactive lipid mediators of the eicosanoid family related to a wide range of biological effects including lipid homeostasis and inflammatory response. C20:4 is essential in many organs such as liver and brain, where it is one of the most abundant fatty acids. In skeletal muscle, C20:4 promotes myocyte growth both *in vitro*^1^ and *in vivo*^2^ through the Akt/mTOR pathway. Mainly esterified into phospholipids, it also exerts a substantial contribution to maintaining membrane fluidity and in cell signalling. The C20:4 content differs between lipid classes, tissues and muscles and is influenced by both the diet and the individual’s genetic background^3,4^. Diets rich in C20:4 or diets producing relatively high levels of linoleic acid (C18:2, *all*-*cis*-9,12–18:2) result in enhanced levels of C20:4 in plasma^2,5^ and muscle^2,6^. Although C20:4 can be taken up from the diet, it can also be synthesised in the animal. There is evidence indicating that the biosynthesis of C20:4 is genetically mediated, notably in pigs, where substantial genetic variation between^4^ and within genetic types^7^ has been reported.

**Figure 1:**
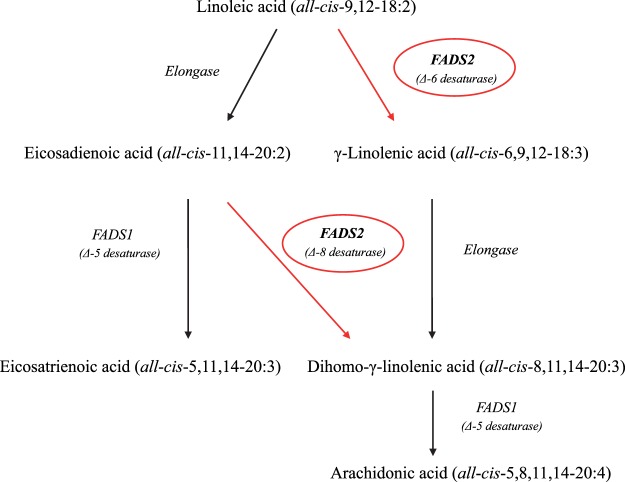
The role of FADS2 in the biosynthesis of arachidonic acid from linoleic acid. The fatty acid desaturase-2 (FADS2, *Δ-6 desaturase*) catalyses the first step for the biosynthesis of arachidonic acid (*all*-*cis*-5,8,11,14–20:4), in which linoleic acid (*all*-*cis*-9-12-18:2) is desaturated to γ-linolenic acid (*all*-*cis*-6,9,12–18:3) and then elongated into dihomo-γ-linolenic acid (*all*-*cis*-8,11,14–20:3). Alternatively, linoleic acid is elongated into eicosadienoic acid (*all*-*cis*-11,14–20:2), which in turn can be either desaturated to eicosatrenoic acid (*all*-*cis*-5,11,14–20:3) via fatty acid desaturase-1 (FADS1, *Δ-5 desaturase*) or to dihomo-γ-linolenic acid (*all*-*cis*-8,11,14–20:3) via FADS2 (*Δ-8 desaturase*). The arachidonic acid is finally synthetized by desaturating dihomo-γ-linolenic acid via FADS1.

The delta-6-desaturase enzyme, encoded by *the fatty acid desaturase-2* (*FADS2*) gene, is responsible for the first and rate-limiting step in the biosynthesis of C20:4 (Fig. 1), where C18:2 is desaturated to γ-linolenic acid (*all*-*cis*-6,9,12–18:3)^8^. The lack of FADS2 leads to obesity resistance and, as reported by Stoffel *et al*.^9^ using auxotrophic mice mutants, it may activate a surrogate reaction in which C18:2 is elongated to eicosadienoic acid (C20:2, *all*-*cis*-11,14–20:2) and then to eicosatrienoic acid (*all*-*cis*-5,11,14–20:3) but not to C20:4. In pigs, *FADS2* is located in chromosome 2 (2:9632454–9667044:-1) as a part of a cluster including *FADS1* and *FADS3*. The genomic structure of the gene is comprised of 12 exons and 11 introns (assembly Sscrofa11.1), which produce three protein-coding splice variants. The activity of FADS2 in the synthesis of long chain fatty acid is enhanced by an alternative transcript of pig *FADS1*^10^, as also reported in baboons and humans^11^. In a recent genome-wide association study with five divergent pig populations, Zhang *et al*.^12^ provide evidence that in Erhualian pigs the region containing *FADS2* was associated with the C20:4 content in muscle. Although an uncharacterised polymorphism in exon 3 of the pig *FADS2* has been associated with C20:4 and intramuscular fat (IMF) content^13^, the sequence variation of *FADS2* has not been otherwise investigated. *FADS2* is a TATA-less gene and such genes are often subjected to complex transcription mechanisms. This makes the promoter region of *FADS2* a sensible location for screening for DNA polymorphisms. The aim of our study was to describe genetic variants in the promoter of the porcine *FADS2* and then to further investigate their association with C20:4 and fat content in the main lipogenic tissues. To this end, we made use of a biorepository of fat, muscle and liver specimens from a high-fat Duroc pig line where at least two relevant genes for fatty acid composition are also segregating^14^^,^^15^.

## Materials and Methods

### Ethics Statement

All pigs used in the study were raised and slaughtered in commercial units following applicable regulations and good practice guidelines on the protection of animals kept for farming purposes, during transport and slaughter. The experimental protocol was approved by the Ethical Committee on Animal Experimentation of the University of Lleida.

### Animals and phenotypes

A total of 1,192 barrows from 159 sires and 590 dams of the same Duroc line were used in this experiment. Pigs were raised in 20 batches between 2002 and 2016 following a similar standard protocol for data recording and tissue sampling^16^. In each batch, pigs were raised from 75 days of age until slaughter at 210 days in the same farm under identical conditions. Pigs had ad libitum access to commercial feed (Esporc, Riudarenes, Girona, Spain). From 160 days of age onwards they were fed a finishing diet including around 6.0% fat (27% C18:2 and 0.3% C20:4 of total fatty acids). All pigs were slaughtered in the same abattoir, where carcass weight and carcass backfat and loin thickness at 6 cm off the midline between the third and fourth last ribs were measured by an on-line ultrasound automatic scanner (AutoFOM, SFK-Technology, Denmark). Immediately after slaughter, samples of semimembranosus (SM, n = 187) muscle, subcutaneous adipose tissue (n = 388) and liver (n = 118) were collected, snap-frozen, and stored at −80 °C. After chilling for about 24 h at 2 °C, samples of the muscles gluteus medius (GM, n = 1,179) and longissimus thoracis (LM, n = 548) were collected, vacuum packaged, and stored at −20 °C. Intramuscular and liver fat content, as well as fatty acid composition, were determined in duplicate by quantitative gas chromatography^17^. The proportion of C18:2, C20:2 and C20:4 were expressed as percentages relative to total fatty acid content (14:0; 16:0; *cis*-9–16:1; 18:0; *cis*-11–18:1; *cis*-9–18:1; C18:2, *all*-*cis*-9,12,15–18:3; 20:0; *cis*-13–20:1; C20:2; and C20:4) and their ratios calculated as indicators of FADS2 activity.

### Genotyping

Genomic DNA was isolated from GM muscle samples using a standard protocol. The proximal promoter of the *FADS2* (~1 Kb) was amplified and sequenced in a subset of 14 pigs with high or low C20:4 content in SM (Supplementary Table S1) with primers and conditions detailed in Supplementary Table S2. An RFLP-PCR genotyping protocol was set up to genotype the rs321384923A > G substitution. PCRs were carried out in 13 μL reactions containing 60 ng of genomic DNA, 1x buffer, 200 nM of dNTP mix, 2 mM of MgCl_2_, 500 nM of each primer and 1 U of Taq polymerase (Bioline). Thermocycling conditions were 95 °C 10 min, 35 cycles of 95 °C 20 sec, 56 °C 20 sec and 72 °C 20 sec finishing with 72 °C 5 min. Ten μl of PCR were digested with *Ava*I (37 °C × 3 h) and solved by electrophoresis in agarose gels. Additionally, two other SNPs known to influence fat content and composition in our resource Duroc line (the *AY487830:g.2228* *T* > *C* SNP at the stearoyl-CoA desaturase (*SCD*) gene on chromosome 14 and the *NM_001024587:g.1987C* > *T* SNP at the leptin receptor (*LEPR*) gene on chromosome 6) were genotyped as described in Ros-Freixedes *et al*.^15^

### *FADS2* expression

Total RNA from 70 SM samples from two batches (AA, n = 14; AG, n = 26; GG, n = 30) and 31 livers from one batch (AA, n = 2; AG, n = 15; GG, n = 14) was isolated with TRI-Reagent (Sigma-Aldrich) following the manufacturer’s indications. Purity of the RNA was assessed by spectrophotometry with a Nanodrop-1000 and the integrity was tested by electrophoresis in agarose gels. *FADS2* and two reference genes, *YWHAZ* and *RPL32*, were analysed by a quantitative PCR assay (Supplementary Table S3). Briefly, 2 μg of total RNA were reverse transcribed using SuperScript IV Reverse Transcriptase (Invitrogen) with oligo-dT and random primers. Real-time PCR assays were carried out in triplicate in 8 μl reactions, containing 1x iTaq Universal SYBR Green Supermix (Bio-Rad), 200 nM of each primer and 3 μl cDNA template diluted 1:30 in water. Cycling parameters were 95 °C for 10 min, 40 cycles of 95 °C for 15 s and 60 °C for 1 min, followed by melt curve analysis. To quantify and normalise the *FADS2* expression data, we used the ΔΔCt method against the geometrical mean of the two reference genes^18^.

### Statistical analyses

The association analysis of *FADS2* genotypes with C18:2, C20:2, C20:4 and their ratios was performed using a mixed model including the batch (20 levels for fatty acids), the *FADS2* genotype (3 levels), the *SCD* genotype (3 levels) and the *LEPR* genotype (3 levels) as fixed effects and the sire and the dam as random effects, with fat content as a covariate (IMF for muscle, backfat thickness for subcutaneous fat and fat content for liver). The same model was used for gene expression (without the covariate) and for carcass traits (with the age at slaughter as a covariate instead of fat content). Additivity was tested replacing the genotype effect by the covariate [1, 0, −1] for the AA, AG, and GG genotypes, respectively. The effects of the *FADS2* genotype and additivity were tested using the F-statistic while the pairwise differences among *FADS2* genotypes were contrasted with the Tukey-HSD test. Results are presented as least-square means ± standard error and were considered statistically significant at P < 0.05. The non-linear relationship of IMF with C20:4 was assessed regressing the reciprocal term of IMF on C20:4. All models were solved using the JMP Pro 12 package (SAS Institute Inc., Cary, NC).

## Results

### Sequence variability at the *FADS2* promoter

A total of 5 SNP polymorphisms and one 12 bp insertion were segregating in the proximal promoter of the pig *FADS2* gene (Supplementary Fig. S1). Among them, three SNPs (rs336076510, rs321384923 and rs331050552 at positions -676, -706 and -798 bp upstream the ATG codon, respectively) were fully linked forming two haplotypes (AAT and GGC). The stability of the two haplotypes was confirmed by genotyping the three SNPs in a subset of 51 pigs evenly distributed across haplotypes (data not shown). The middle SNP, rs321384923A > G substitution, was selected for further analysis as it modified a potential retinoic acid/oestrogen related receptors (TGCCCG) binding site while no potential transcription factor binding sites were detected in the other two SNP sites. While human and rat *FADS2* expression responds to oestrogen hormone^19^ and vitamin A^20^, identification of causal mutations has been hindered by the presence of clusters of polymorphisms in strong linkage disequilibrium both upstream and downstream of the translation start site^21^.

### *FADS2* rs321384923 genotype frequencies

The frequencies of the *FADS2* rs321384923 genotypes by *SCD* and *LEPR* genotypes are given in Supplementary Table S4. The A allele was the minor allele (frequency of 30.9%). The *g.2228* *T* > *C SCD* and the *g.1987C* > *T LEPR* SNPs were both segregating at intermediate frequencies (46.1% and 43.5% for the T allele, respectively). All possible genotypes for the three SNPs were observed and, as expected for genes in different chromosomes, they were in linkage equilibrium (r^2^ < 0.005, for all pairwise linkage disequilibrium between SNPs).

### *FADS2* genotype and *FADS2* expression

**Figure 2:**
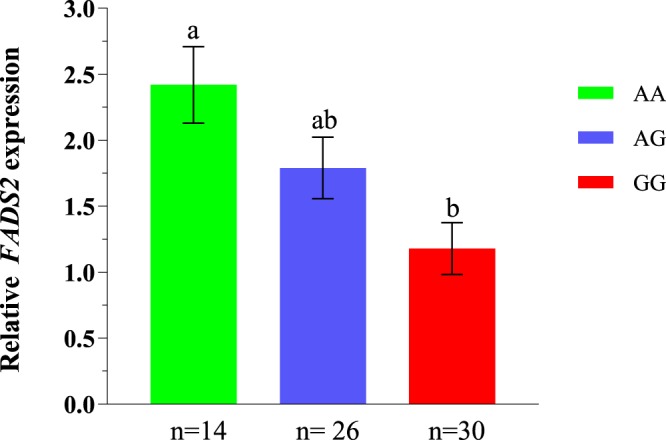
Relative *FADS2* mRNA expression in muscle by rs321384923 genotype. The *FADS2* gene expression in the semimembranosus muscle was around two-fold higher for the AA genotype as compared to the GG genotype. The number of pigs (n) per genotype ranged from 14 to 30. Error bars represent standard errors. Means with different superscripts differ significantly (P < 0.05).

*FADS2* expression was determined in SM on pigs of the three *FADS2* rs321384923 genotypes (Fig. 2). The expression analysis was only performed in SM, since it was not possible to obtain GM and LM samples immediately after slaughter. The relative gene expression of *FADS2* in muscle was 2-fold higher in the AA genotype as compared to the GG genotype (2.34 vs 1.10, P < 0.01). As evidenced by the allele substitution effect (0.63 ± 0.18, P < 0.01), heterozygous pigs displayed intermediate levels of gene expression. Results of *FADS2* expression in liver confirmed the same trend, with pigs carrying the A allele (AA and AG) showing higher expression than the GG pigs (2.83 vs 1.38, P < 0.05). From this foundation, we proceed to explore the possible functional consequences caused by the higher expression of the A allele at the porcine *FADS2*.

### *FADS2* genotype and arachidonic acid

The effect of the *FADS2* rs321384923 was first assessed in GM (Table 1). The results are presented adjusted for IMF, but those unadjusted led to similar conclusions. AA pigs had 12.5% more C20:4 and 6.1% less C20:2 in GM than GG pigs. As suggested by the expression data, the *FADS2* SNP displayed an additive behaviour, with a G to A allele substitution effect of 0.09 ± 0.02, for C20:4 (P < 0.001), and -0.13 ± 0.03, for C20:2 (values x 10; P < 0.001). The same trend was observed when fatty acids were quantitatively expressed in mg/g of muscle. These values, although only accounting for about 2% of the total variance of these two fatty acids (2.2% for C20:4 and 1.7% for C20:2), provide support to the hypothesis that there exists genetic variation in the promoter region of the *FADS2* that impacts n-6 fatty acid biosynthesis in pigs. This was confirmed by analysing the indicator ratios of FADS2 activity (Table 1). Regarding the C20:4 to C18:2 ratio, which can be interpreted as the overall efficiency of transforming C18:2 to C20:4, AA pigs were 12.0% more efficient than GG pigs. Interestingly, for the alternative route that converts C18:2 into C20:2 (C20:2/C18:2 ratio), AA pigs were 2.9% less efficient than GG pigs. However, since FADS2 also acts in the desaturation pathway from C20:2 to C20:4, a supplementary effect of the *FADS2* SNP on C20:4 is expected to occur over this route, with the A allele further enhancing the synthesis of C20:4 and the G allele accumulating more C20:2. This effect was highlighted by the relatively greater differences by genotype for the C20:4/C20:2 ratio, which was 17.8% higher in AA pigs than in GG pigs, explaining up to 5.8% of the total variance of the ratio. On the other hand, C18:2, the primary substrate in the endogenous metabolism of C20:4, should decrease with increased FADS2 activity. We only were able to detect this effect when C18:2 was expressed in mg/g of muscle instead of as a percentage of total fatty acids. Then, as expected, AA pigs showed the lowest value of C18:2 (15.4 mg, 16.0 mg and 16.0 mg for AA, AG and GG, respectively, P < 0.05).

**Table 1:** Effect of the *FADS2* rs321384923 genotype on n6-fatty acid composition.

Trait	P-value^a^	*FADS2* ^b^		
		AA	AG	GG
Backfat, mm	0.99	26.3 ± 0.4	26.3 ± 0.3	26.3 ± 0.2
IMF, % dry matter	0.10	18.0 ± 0.5	18.7 ± 0.3	19.1 ± 0.3
C18:2, %	0.13	10.06 ± 0.13	10.28 ± 0.07	10.33 ± 0.07
C20:2, % (x10)	<0.001	4.65 ± 0.07^a^	4.85 ± 0.04^b^	4.95 ± 0.04^b^
C20:4, %	<0.001	1.62 ± 0.04^a^	1.55 ± 0.02^a^	1.44 ± 0.02^b^
C20:4/C18:2 (x10)	<0.001	1.59 ± 0.03^a^	1.52 ± 0.02^a^	1.42 ± 0.02^b^
C20:2/C18:2 (x100)	0.004	4.70 ± 0.04^a^	4.77 ± 0.02^a^	4.84 ± 0.02^b^
C20:4/C20:2	<0.001	3.51 ± 0.07^a^	3.25 ± 0.04^b^	2.98 ± 0.04^c^

As compared to the GG pigs, the AA pigs showed a higher content of arachidonic acid (C20:4) and a lower content of eicosadienoic acid (C20:2) in muscle because they were more efficient transforming linoleic acid (C18:2) into C20:4. Subcutaneous fat was measured in terms of backfat thickness and intramuscular fat was determined in gluteus medius muscle. The proportion of each fatty acid is expressed as a percentage relative to total fatty acid content and, as well as ratios, adjusted for intramuscular fat (IMF) content. ^a^P-value associated with the effect of the *FADS2* genotype; ^b^Pairwise comparisons of *FADS2* genotypes. ^c^Within row, means with different superscripts differ significantly (P < 0.05).

**Figure 3:**
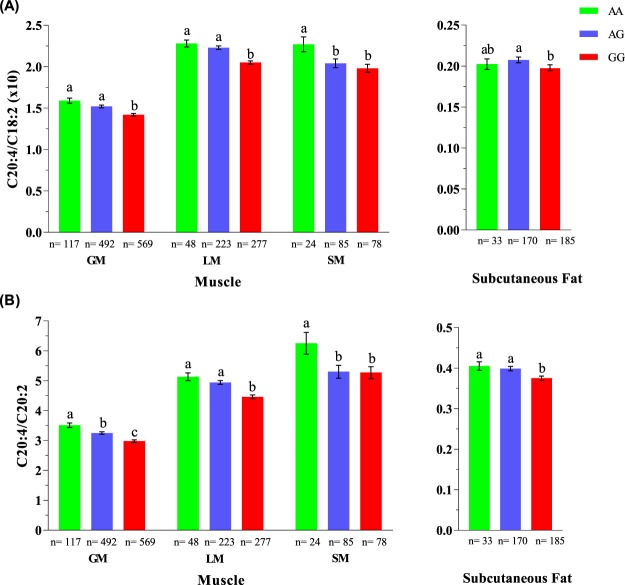
Efficiency of arachidonic acid biosynthesis by *FADS2* rs321384923 genotype in muscle and subcutaneous fat. (**A**) The AA genotype of *FADS2* was more efficient than the GG genotype in transforming linoleic acid (C18:2) into arachidonic acid (C20:4) both in muscle (GM: m. gluteus medius; LM: m. longissimus thoracis muscle; and SM: m. semimembranosus muscle) and in subcutaneous fat, around 12% and 2%, respectively. As a result, (**B**) C20:4 to eicosadienoic acid (C20:2) ratio in muscle and in subcutaneous fat was, respectively, 18% and 8% greater in AA pigs as compared to GG pigs. The number of pigs (n) genotyped per tissue and genotype ranged from 24 to 569. Error bars represent standard errors. Within tissue, means with different superscripts differ significantly (P < 0.05).

The association of *FADS2* rs321384923 genotypes with fatty acid composition was investigated in two other muscles (LM and SM), subcutaneous fat and liver. The effect of *FADS2* SNP in LM and SM were in line with those observed in GM, particularly for the C20:4 to C18:2 and C20:4 to C20:2 ratios (Fig. 3). As compared to GG pigs, AA pigs had a greater proportion of C20:4 in relation to C18:2, both in LM (2.28 vs 2.05, values x10) and in SM (2.27 vs 1.98, values x10; Fig. 3A) and to C20:2, also both in LM (5.13 vs 4.46) and in SM (6.25 vs 5.27, Fig. 3B). The C20:4 to C18:2 and the C20:4 to C20:2 ratios gave similar results in subcutaneous fat, albeit around twenty times smaller in magnitude (Fig. 3B). However, we did not find differences across genotypes for these two ratios in liver (Supplementary Fig. S2). Regarding C20:4 content, the effect of the *FADS2* genotype in LM was fully consistent with the results in GM, with AA pigs performing better than GG pigs (1.84% vs 1.62%, with a substitution effect of 0.12 ± 0.02, P < 0.001). Likely due to the limited data size, this effect was not evident in SM (3.12%, for AA, and 2.74%, for GG, with a substitution effect of 0.13 ± 0.11, P = 0.24). No difference between *FADS2* genotypes was detected for C18:2 and C20:2 in both LM and SM. The results in LM and SM were the same when individual fatty acids were expressed in mg/g of muscle.

### *Relationship of FADS2 and LEPR* genotypes with fat content

**Figure 4:**
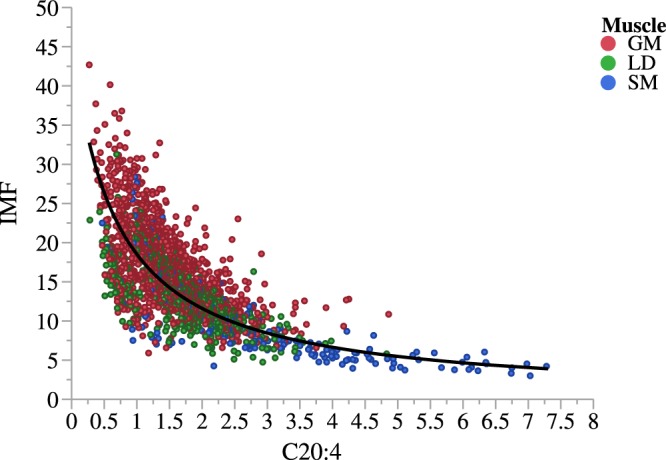
Relationship of arachidonic acid in muscle with intramuscular fat content. The arachidonic acid (C20:4) content, expressed as a percentage of total fatty acids, is negatively related to intramuscular fat (IMF^-1^ = 0.0218 + 0.0323*C20:4; R^2^:0.66). The regression was obtained across muscles using 1,912 datapoints from gluteus medius (GM, n = 1,177), longissimus thoracis (LM; n = 548) and semimembranosus (SM; n = 187) muscles. The GM showed the lowest C20:4 content (raw mean of 1.47%, 1.72% and 2.95%, for GM, LM and SM, respectively) and the highest level of IMF (raw mean of 17.2%, 13.3% and 9.6% for GM, LM and SM, respectively).

We did not find evidence of association of *FADS2* rs321384923 with carcass weight and lean percentage and, as a result, with lean weight (Table S5). The *FADS2* genotype was not associated with IMF either (Table S5), although the A allele showed a negative trend towards decreasing IMF in GM, where the allele substitution effect was −0.49 ± 0.23 (P < 0.05). This finding contrasts with the clear-cut negative relationship between C20:4 and muscle fat content (Fig. 4). However, two overlapping phenomena should be considered when accounting for C20:4 in relation to total fatty acid content: the efficiency in transforming C18:2 into C20:4 and the dilution of C20:4 as overall endogenous fat synthesis progresses, two effects which in turn are not fully independent. As indicated by the covariate adjustment, IMF showed a negative relationship not only with all investigated n-6 fatty acids (beta: −0.07% ± 0.01, P < 0.001, for C20:4) but also with all proxy ratios (beta: −0.04 ± 0.01, P < 0.001, for C20:4/C18:2, values x10) except C20:2/C18:2 (beta: 0.06 ± 0.01, P < 0.001, values x 100), thereby indicating that increased fat content, in addition to diluting C20:4, involves a decline in the biosynthesis efficiency of C20:4. Both effects are taken into account by adjusting *FADS2* genotype comparisons for fat content. Therefore, the differences in C20:4 between *FADS2* genotypes should be attributed to differential efficiency performance.

To validate the specificity of the *FADS2* genotypes on C20:4 biosynthesis efficiency we made use of the *LEPR* g.1987C > T SNP as an internal control gene for fatness, provided that this polymorphism, which co-segregates with FADS2 SNP in this population, is known to affect lipid accumulation. The *LEPR* TT pigs used here produced around 5% and 11% more backfat and IMF, respectively, than *LEPR* CC pigs (Table 2) at no significant change in carcass weight. Whether adjusted for IMF or not, the results for the *LEPR* SNP on C20:4 were in line with the expected from Fig. 4, with the C allele affecting negatively IMF and positively C20:4 (Table 2). However, in contrast to the *FADS2* SNP, the favourable effect of the *LEPR* C allele on C20:4 was accompanied by concomitant increases in C18:2 and C20:2. Hence, the *LEPR* SNP had no effect on the ratios associated with FADS2 activity (C20:4/C182, C20:2/C18:2 and C20:4/C20:2) when adjusting for IMF (Table 2). The dissimilar behaviour of *FADS2* and *LEPR* SNPs in relation to these ratios substantiates the two paths by which C20:4 can be modified. Thus, while the effect of the *LEPR* SNP on C20:4 is to a great extent a matter of scale, a result of variations in fat content, the effect of the *FADS2* polymorphism is based on changes in efficiency, which does not necessarily mean variations in fat content. No interaction between *LEPR* and *FADS2* genotypes was observed for these fatty acids. The effect of the *SCD* genotype was neutral for n-6 fatty acid composition and fat content.

**Table 2:** Effect of the *LEPR g.1987C* > *T* SNP genotype on n6-fatty acid composition.

Trait	P-value^a^	*LEPR* ^b^		
		CC	CT	TT
Backfat, mm	0.003	25.8 ± 0.3^a^	26.1 ± 0.3^a^	27.0 ± 0.3^b^
IMF, % dry matter	<0.001	17.9 ± 0.3^a^	18.1 ± 0.3^a^	19.8 ± 0.4^b^
C18:2, %	<0.001	10.49 ± 0.09^a^	10.29 ± 0.08^a^	9.89 ± 0.10^b^
C20:2, % (x10)	<0.001	4.94 ± 0.05^a^	4.84 ± 0.04^a^	4.65 ± 0.06^b^
C20:4, %	0.01	1.59 ± 0.03^a^	1.54 ± 0.02^ab^	1.48 ± 0.03^b^
C20:4/C18:2 (x10)	0.44	1.53 ± 0.02	1.50 ± 0.02	1.49 ± 0.02
C20:2/C18:2 (x100)	0.85	4.77 ± 0.03	4.76 ± 0.03	4.78 ± 0.03
C20:4/C20:2	0.64	3.28 ± 0.05	3.24 ± 0.04	3.21 ± 0.06

As compared to TT pigs, the CC pigs showed a higher content of arachidonic acid (C20:4) and eicosadienoic acid (C20:2) in muscle because they were fatter and not because they were more efficient transforming linoleic acid (C18:2) into C20:4 and C20:2. Subcutaneous fat was measured in terms of backfat thickness and intramuscular fat was determined in gluteus medius muscle. The proportion of each fatty acid is expressed as a percentage relative to total fatty acid content and, as well as ratios, adjusted for intramuscular fat (IMF) content. ^a^P-value associated with the effect of the *LEPR* genotype; ^b^Pairwise comparisons of *LEPR* genotypes. Within row, means with different superscripts differ significantly (P < 0.05).

## Discussion

We report here a SNP in the promoter region of the pig *FADS2* (rs321384923) with effect on the desaturation pathways leading to C20:4 biosynthesis. The polymorphism is a tagging SNP for a 3-SNP haplotype in the *FADS2* promoter and it is situated -706 bp upstream from the ATG codon and -294 bp upstream from the start of transcription of the closest *FADS2* transcript (ENSSSCT00000014289.3). Two previous genome-wide association studies have identified markers around this haplotype region associated with *FADS2* expression and fatty acid metabolic traits. In the first one, in an Iberian x Landrace backcross, Revilla *et al*.^22^ found that the most significant cis-SNP for *FADS2* gene expression (rs81474400) was located 230 Kb upstream of the *FADS2*. In this study, the three SNPs of the haplotype were found to be segregating in the Iberian founders, but no association was observed between the most proximal of them (rs331050552) and *FADS2* mRNA expression in the backcrossed pigs. The low frequency of the minor allele and/or different haplotype structure in the backcrossed individuals could have interfered with the expected results in an experimental population of limited size. In the second genome-wide association study, a SNP (rs81360272) located in the fifth intron of *FADS2*, was reported to be associated in Erhualian pigs with proxy ratios of FADS2 activity^12^. SNPs from both studies are from the Illumina’s pig genotyping array and encompass a region containing at least four additional SNPs from this array. Using data on 272 Duroc pigs from our line genotyped with this chip, we found that these two SNPs were in low linkage disequilibrium with our tag SNP (r^2^ = 0.10–0.15) and had no effect on C20:4 and associated ratios. In contrast, the two nearest upstream SNPs to our haplotype (rs343441264 and rs81360470) were almost fully linked with our tag SNP (r^2^ = 0.88–0.92) and parallel their effects. Overall, this genomic pattern would confirm that our tag SNP is capturing a functional variant in the promoter region of *FADS2* influencing C20:4 content in pigs.

The presence of the A allele of the rs321384923 SNP additively enhances *FADS2* expression and, as a result, the desaturase activity in both muscle and subcutaneous fat. FADS2 is the rate limiting enzyme in the conversion of essential fatty acids C18:2 and α-linolenic to long-chain polyunsaturated fatty acids. The knock out of this gene results in mice lacking polyunsaturated fatty acids beyond eicosatrienoic acid^23^ (*all*-*cis*-5,11,14–20:3; Fig. 1), indicating that there are no other enzymes with a redundant activity. FADS2 participates in two well-characterized steps in the biosynthesis of n-6 polyunsaturated fatty acids: the desaturation of (i) C18:2 to γ-linolenic acid (*all*-*cis*-6,9,12–18:3) and (ii) C20:2 to dihomo-γ-linolenic acid (*all*-*cis*-8,11,14–18:3). These are critical steps to produce C20:4. Thus, the presence of the more active A allele accelerates the production of C20:4 through both routes, as seen by significant changes in the C20:4/C18:2 and C20:4/C20:2 ratios, resulting in 12–14% more C20:4 in the three muscles tested (GM, LM and SM) regardless of IMF. Furthermore, this was a consistent additive effect, paralleling the gene expression results. In agreement with this, there was a small correlated decrease in the amount of C18:2 in the genotypes carrying the A allele. These results are in line with previous findings^9^ indicating that a lack of FADS2 triggers an alternate reaction where C18:2 is diverted to C20:2 instead of *all*-*cis*-6,9,12–18:3, the first intermediate fatty acid in the canonical endogenous synthesis of C20:4. In the absence of FADS2, C20:2 cannot be transformed to *all*-*cis*-8,11,14–20:3, the last precursor of C20:4, and thus it is further desaturated via FADS1 to *all*-*cis*-8,11,14–20:3, an aberrant fatty acid which is incorporated as a surrogate of C20:4 in the diacylglycerol-backbone of membrane phospholipids. Unfortunately, we have no available data to test whether *all*-*cis*-8,11,14–20:3 declines as expected with the presence of the A allele.

Despite the fact that, in pigs, liver and subcutaneous fat express 8–10 times more *FADS2* than muscle^10^, the effect of the *FADS2* rs321384923 on the accumulation of C20:4 is more evident in muscle than in subcutaneous fat and liver. Apart from having more statistical power in muscle, the functional approach in this tissue can be more accurate for the reason that it shows a relatively higher de novo fatty acid synthesis^24^. In this regard, the case of the Duroc pigs is particularly interesting because they present a higher proportion of C18:2 in IMF relative to other breeds^3^. Moreover, an alternative transcript of *FADS1*, particularly enriched in subcutaneous fat and liver^10^, has been shown to regulate FADS2 activity in humans^11^. In this line, in a previous expression genome-wide association study^22^, the correlation between *FADS1* and *FADS2* mRNA expression was higher in liver than in subcutaneous fat (r = 0.92 and 0.63, respectively) while *FADS2* expression showed the lowest correlation (r = 0.23) between these two tissues. Thus, the interaction between these two genes and tissue-specific mechanisms of regulation is a question worth exploring in the future.

Many human and mouse studies positively correlates Δ-6 activity with metabolic syndrome, insulin resistance and obesity (reviewed in Naughton *et al*.^25^). Diets rich in C18:2 result in an increase in C20:4, which can then be converted into prostacyclins and endocannabinoids, both of them with a strong pro-adipogenic activity^25^. Indeed, although many of these studies report weight gain and increased adipose inflammation, the final outcome is highly dependent on the whole diet and other external factors such age or physical activity. Some research even showed that dietary supplements with C18:2 increase lean mass^26^. In our study with pigs none of the carcass traits analyzed were affected by the higher FADS2 activity of the A rs321384923 allele. Even though C20:4 was negatively correlated with carcass weight (r = −0.39, p < 0.01, for GM) and positively with lean content (r = 0.22, p < 0.01, for GM), the effect of the *FADS2* SNP on n6-fatty acid composition did not *per se* modify these traits. Our results also indicate that, although unevenly across muscles, the tag SNP could exert some influence on IMF, especially in muscles displaying lower C20:4 content (i.e. GM, with 15% and 50% less C20:4 than LM and SM, respectively; Fig. 4). Using data from four commercial genetic types, Renaville *et al*.^13^ found that a SNP in the exon 3 of *FADS2* was associated with contents of C20:4 and IMF, but only in LM and not in muscle biceps femoris. In line with our results, the favorable effect on IMF was associated with the negative allele for C20:4 and evidenced in the muscle with the lowest C20:4. Moreover, the potential distinct effect of the *FADS2* SNP on IMF in different muscles could be attributed to the differential partitioning of C20:4 between neutral lipids and phospholipids across muscles. Thus, Wood *et al*.^3^ found that the proportion of C20:4 in phospholipids as compared to neutral lipids was higher in LM (around 50 times) than in psoas major (around 10 times), even when both muscles were compared at a similar level of IMF. In a limited subset of pigs, we obtained that in GM C20:4 was around 35 times more abundant in phospholipids than in neutral lipids. These results indicate that the ability of C20:4 to get incorporated into membrane phospholipids, and likely to mediate in cell signaling events, could be muscle-specific. Results in mice evidenced that FADS2 deficiency alters the membrane phospholipidomic profiling, affecting the maturation of transcription factor sterol-regulatory- element-binding protein and therefore lipid homeostasis^9^. In this sense, an interesting piece of research to address the nuances of functionality of C20:4 in pig muscle is to determine how differently the *FADS2* genotypes affect phospholipid and neutral lipid fatty acid composition and whether their allocation relates with fat content. IMF is a relevant trait for the pig industry in general, but particularly in Duroc lines used in premium quality meat markets, where pigs are raised to display a high level of IMF. For this reason, rs321384923 cannot be discarded as a candidate marker to increase IMF without altering lean weight.

In line with findings in humans^27^, our results confirm that the porcine *FADS2* is subjected to functional genetic variation while providing evidence that the rs321384923 SNP in its promoter region impacts gene expression. We showed that there is an haplotype tagging SNP in the promoter region of *FADS2* that results in a more efficient transformation of C18:2 into C20:4. However, we were not able to observe any consistent implication of this on the traits usually selected for in pig populations. Evidence in humans indicates that fatty acid desaturases affect plasma and tissue lipid profiles and therefore associated disease risk factors. Recent findings in humans suggest that *FADS* genes have been subjected to strong positive selection in response to C18:2 consumption and that this event is not neutral in relation to plasma cholesterol levels^28^ and to chronic and inflammatory disorders^27^. Further studies are needed to identify the molecular mechanisms by which variation in *FADS2* modulates gene expression and functional phenotypes. In this regard, the present work confirms that selected pig populations can be an interesting genetic resource.

## Electronic supplementary material

Supplementary Information

## Data Availability

All data supporting the findings of this study are available within the article and Supplementary Information, or are available from the corresponding author upon reasonable request.
