# Supplementary material for: A polymorphism in the fatty acid desaturase-2 gene is associated with the arachidonic acid metabolism in pigs
Source: Sci Rep. 2018 Sep 25;8:14336. doi: 10.1038/s41598-018-32710-w (PMC6156218; doi:10.1038/s41598-018-32710-w)

**Supplementary Information**

**A polymorphism in the fatty acid desaturase-2 gene is associated with the arachidonic acid metabolism in pigs**

Sofia Gol^1^, Ramona N. Pena^1^, Max F. Rothschild^2^, Marc Tor^1^, and Joan Estany^1^

^1^Universitat de Lleida - Agrotecnio Center, Department of Animal Science, 25198, Lleida, Catalonia, Spain.

^2^ Iowa State University, Department of Animal Science, Ames, IA, 50011 USA

**Table S1. Description of the animals used to sequence the *FADS2* promoter**. From the resource Duroc population, a subset of 14 animals from two batches were selected based on their intramuscular fat (IMF, in % dry matter) and arachidonic acid (C20:4, in % of total fatty acids) content in the gluteus medius muscle (GM), longissimus thoracis (LM) and semimembranosus muscle (SM). On average, pigs in group A had about twice as much C20:4 than pigs in Group B.

|  |  |  | IMF, % | | |  | C20:4, % | | |
| --- | --- | --- | --- | --- | --- | --- | --- | --- | --- |
| Group | No of pigs |  | GM | LM | SM |  | GM | LM | SM |
| A | 7 |  | 13.4 | 9.6 | 5.6 |  | 2.5 | 3.0 | 5.9 |
| B | 7 |  | 20.7 | 17.2 | 13.3 |  | 1.3 | 1.3 | 2.3 |

**Table S2. Primers (A), reagents (B) and (C) cycling PCR conditions used to sequence the pig *FADS2* proximal promoter.**

**A**. **Primers used to amplify the proximal promoter of the pig *FADS2* gene.**

| Primer | Sequence (5’ 🡪 3’) | Tm | Expected size |
| --- | --- | --- | --- |
| FADS2_PF1 | ACCCCCACCTTTATTTCCTG | 59.7 ºC | 1097 bp |
| FADS2_PR1 | TTGCTTTCGGCTTTTGTCTT | 55.7 ºC |

**B**. **Reagents used to set up the *FADS2* promoter PCR.**

| Reagents | Volume per PCR reaction |
| --- | --- |
| H_2_O | 9.5 µl |
| 10x Buffer | 1.5 µl |
| 50 mM MgCl_2_ | 0.75 µl |
| 5 mM dNTP | 0.6 µl |
| Primer mix (Fw+Rv) 10 µM each | 0.6 µl |
| Taq polymerase, Bioline (5 U/µl) | 0.06 µl |
| Genomic DNA (30 ng/µl) | 2 µl |
| Total | 15 uL |

**C**. **Cycling conditions used to PCR the pig *FADS2* promoter.**

| PCR Program | | | | |
| --- | --- | --- | --- | --- |
|  | | Temperature |  | Time |
| Initial DNA denaturing | | 94ºC |  | 4 min |
| 35 cycles | DNA denaturing | 97ºC |  | 15 sec |
|  | Primer annealing | 60ºC |  | 60 sec |
|  | Extension | 72ºC |  | 90 sec |
| Final extension | | 72ºC |  | 5 min |

**Table S3**. **Primers used to analyse *FADS2* gene expression.**

| Primer | Sequence (5’ 🡪 3’) | Tm | Expected size |
| --- | --- | --- | --- |
| qFADS2_F | GCTGGATTCCAACCCTCATG | 57.9 ºC | 56 bp |
| qFADS2_R | AGCCTGGGCCTGAGAGGTA | 59.8 ºC |
| qYWHAZ_F | TGATGATAAGAAAGGGATTGTGG | 59.4 ºC | 134 bp |
| qYWHAZ_R | GTTCAGCAATGGCTTCATCA | 61.3 ºC |
| qRPL32_F | CACCAGTCAGACCGATATGTCAA | 61.1 ºC | 70 bp |
| qRPL32_R | CGCACCCTGTTGTCAATGC | 61.1 ºC |

**Table S4. Genotype distribution of the *FADS2* single nucleotide polymorphism rs321384923 A>G.** *FADS2* genotypes are presented across stearoyl-CoA desaturase (*SCD*, *AY487830:g.2228T>C*) and leptin receptor (*LEPR*, *NM_001024587:g.1987C>T*) genotypes. The *SCD* and *LEPR* SNPs, which have been shown to exert a substantial influence on fatty acid composition, were also segregating in the resource Duroc pig line used in this study. A total of 1,192 samples have been genotyped for the three polymorphisms.

| ***FADS2*** | | | | | | | | | | | | |
| --- | --- | --- | --- | --- | --- | --- | --- | --- | --- | --- | --- | --- |
|  |  | AA (n=120) | | |  | AG (n= 497) | | |  | GG (n=575) | | |
|  |  | ***LEPR*** | | |  | ***LEPR*** | | |  | ***LEPR*** | | |
| ***SCD*** |  | CC | CT | TT |  | CC | CT | TT |  | CC | CT | TT |
| CC |  | 14 | 10 | 4 |  | 61 | 73 | 33 |  | 55 | 74 | 30 |
| CT |  | 21 | 30 | 22 |  | 78 | 103 | 39 |  | 93 | 134 | 57 |
| TT |  | 11 | 5 | 3 |  | 23 | 61 | 26 |  | 34 | 77 | 21 |

**Table S5. Carcass weight and composition of pigs by *FADS2*** ***rs321384923* genotype.** There was no evidence that the A allele at the *FADS2* genotype had a consistent impact on growth, lean content and fat distribution. ^1^P-value associated with the effect of the *FADS2* genotype; ^2^ Pairwise comparisons of *FADS2* genotypes.

|  |  | |  | *FADS2* genotype^2^ | | | | |
| --- | --- | --- | --- | --- | --- | --- | --- | --- |
| Trait |  | P-value^1^ |  | AA |  | AG |  | GG |
| Carcass weight, kg |  | 0.14 |  | 96.4 ± 1.0 |  | 98.3 ± 0.6 |  | 97.6 ± 0.5 |
| Backfat thickness, mm |  | 0.99 |  | 26.3 ± 0.4 |  | 26.3 ± 0.3 |  | 26.3 ± 0.2 |
| Loin thickness, mm |  | 0.21 |  | 43.3 ± 0.8 |  | 44.5 ± 0.5 |  | 43.7 ± 0.4 |
| Lean, % |  | 0.83 |  | 40.9 ± 0.5 |  | 41.3 ± 0.3 |  | 41.1 ± 0.3 |
| Lean weight, kg |  | 0.11 |  | 39.0 ± 0.6 |  | 40.2 ± 0.3 |  | 39.8 ± 0.3 |
| Intramuscular fat, % dry matter |  |  |  |  |  |  |  |  |
| m. gluteus medius |  | 0.10 |  | 18.0 ± 0.5 |  | 18.7± 0.3 |  | 19.1 ± 0.3 |
| m. longissimus thoracis |  | 0.82 |  | 14.1 ± 0.5 |  | 13.7 ± 0.3 |  | 13.8 ± 0.3 |
| m. semimembranous |  | 0.80 |  | 10.3 ± 1.1 |  | 10.6 ± 0.6 |  | 10.1 ± 0.6 |
| Liver fat content |  | 0.12 |  | 15.1 ± 0.9 |  | 15.9 ± 0.4 |  | 14.8 ± 0.4 |

**Figure S1**. **Sequence of the pig *FADS2* proximal promoter**. The sequence corresponding to the primers used for sequencing is underlined and highlighted in yellow. In bold, coding region. The arrow indicates the start of the first exon in the closest transcript described in Ensembl Sscrofa 11.1 (ENSSSCT00000014289.3). Positions are referred to the ATG codon. In pink, polymorphisms identified during the sequencing process. The SNP selected for this study correspond to rs321384923.

AGGCCTATCCTTTTGCT**ACCCCCACCTTTATTTCCTG**GAGTCTCTACAGCATCACTGAACA -1111

ATTGATGGTATGAGGTCCCGT**ATTGC**TTCCATGTTCTCCCTCTCCCAACTAGGTTGTGAGA -1050

TTCCTGAGTTCAGGGCTAGAACCAAGTCTTGTTCACCCTGGGCCCCCCCACTCTCCCCACC -989

ACGGCGCCCCCCTCCTCCCACCACCATGGCGCCTAACACAGGAAAGCACATCAGAGGTTCT -927

**SNP1 rs344625804**

GCAATTTTCTCCTAAGATCCACTGATTACAGGCTTCAGAGTC**Y**TGAGCCGTCGGGAGGAGG -866

GCTCCTTTCCGAACCAGGGAGGCTGCAGGAGGGCGCCAATGGGGTAAGCGCGGACAATGCG -805

**SNP2=** **rs336076510r**

GATCCT**R**AAGTCTCGATTCCAGCGGGTCAGGGACGGGCGCCACCTATCCAGGCCGGTCCCA -744

**SNP3 rs321384923=rs321384923**

GAGTGGAACTCGCCAGCCCCCCAGCTCCGCGCTGCCC**R**AGCACCCGCCAGCCTGTCTTCCG -683

**SNP4=** **rs331050552** **SNP5**

GCCGGT**Y**GCCACCGTAAAGCAAGCGGGGACCTCTGGGCGCCAGC**W**TCAGGTGCAAACCCCG -622

GAAGCGCGGGCGATGGGGGCGCGCGCACGCCAAGGAACTCAGCCACCGCCCCCTCTCGCGG -561

GCCGCGCTCCCCTGAGATCCCCTCCCGCGGCGCGGCGCCGGAGCGGGGGCGGGAGGAGTTC -500

GGACACGTAACCTGCCTCCCTGCCTGGCTCGACTCCGCGTGGGCGGGCAGGCGGGGGAGCC -439

GGGGACCGCTGCTCCAGCCCGCTGGCCTTCGAAAGATCCTCCTGGGCCAATGGCAGGCGGG -378

CGACGCGCCCGGATTGGTGCAGAAGCTCTGCTGATCACTGTGGAAACCCAGGCGGAGGGGA -317

ACGCGGGAGGATGCAGAGCCCTGGGCGGGGGGAGTCGGAGGGGCGGGCACAGGAGGCCTGG -256

**INSERTION 12bp**

AGGCCCTGAGCCTACCGGGGAGTTTTAGCT◆GGAGGCAAAAGTCCATTGCGGGCTGGCGG -197

GCGGAGGGAGGGGCGGAGGGAGGGGACCGTTTGGGGCCACTGGGAAGCCTGGAGGAAAGGC -136

AAGGATACTCCCGAGCGGAGGCGAGGAGGCTGGGGGAGGGGGCGCGGTGGGAGGAGGAGAA -75

GACAAAAGCCGAAAGCAAGGAGGGCCCGAGCGGCACAGACCGCAGTGCACCGGGCAACCTG -14

GTCAGCGGGCAGC**ATGGGGAAGGGGGGGAACCAGGGCGAGGGGGCCACCGAGCGCGAGGCC** +48

**Figure S2**. **Efficiency of arachidonic acid biosynthesis by *FADS2* genotype in liver.** The presence of the A allele was not seen to be associated neither with (**A**) the arachidonic acid (C20:4) to linoleic acid (C18:2) ratio nor with (**B**) C20:4 to eicosadienoic acid (C20:2) ratio in the liver. Error bars represent standard errors. Within trait, means with the same superscript do not differ significantly (P<0.05).


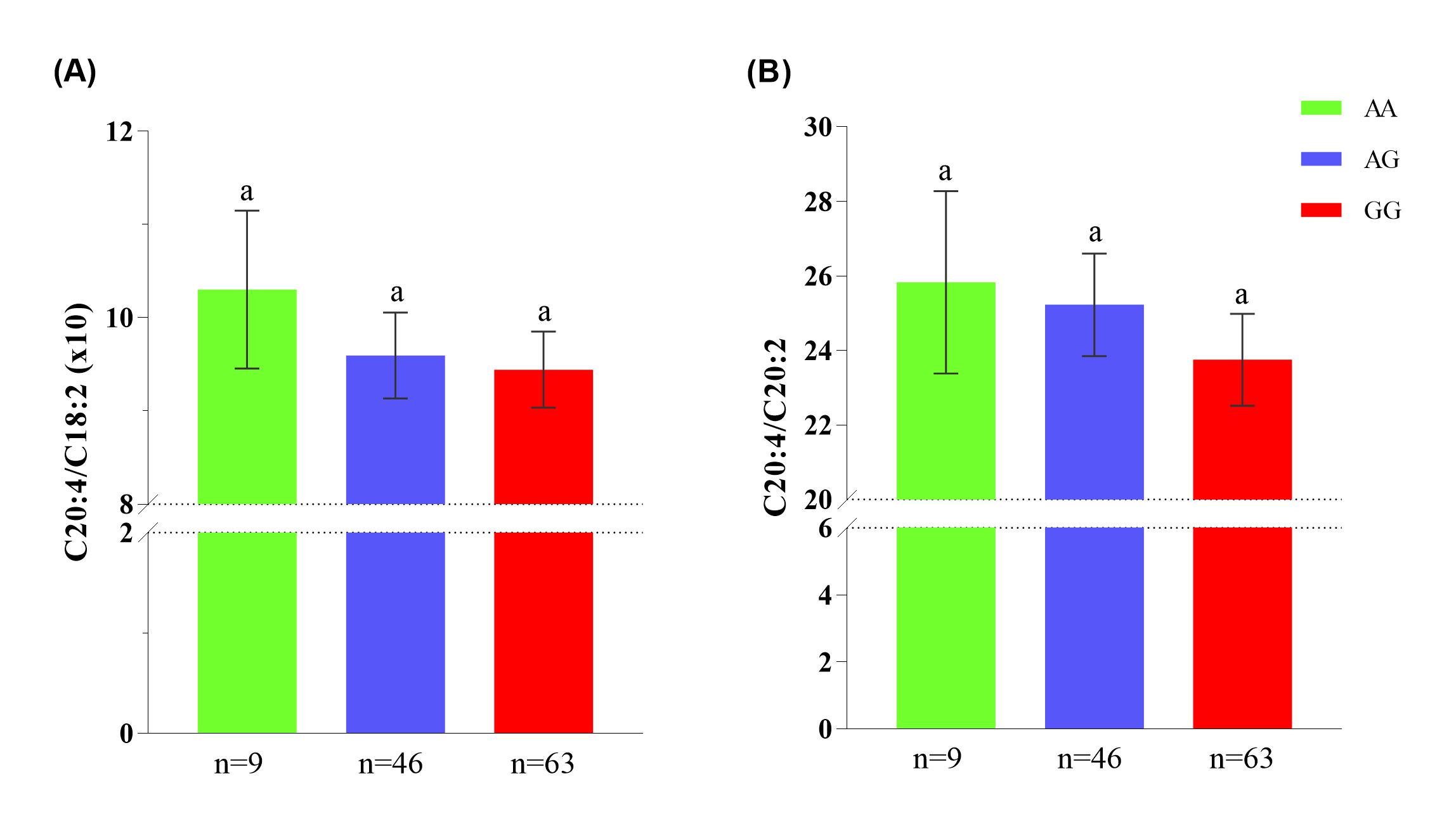

Supplement: Supplementary file 1 — Supplementary Information [file 41598_2018_32710_MOESM1_ESM.doc]
